# Current State of and Needs for Hepatitis B Screening: Results of a Large Screening Study in a Low-Prevalent, Metropolitan Region

**DOI:** 10.1371/journal.pone.0092266

**Published:** 2014-03-24

**Authors:** Julie Bottero, Anders Boyd, Maud Lemoine, Fabrice Carrat, Joel Gozlan, Anne Collignon, Nicolas Boo, Philippe Dhotte, Brigitte Varsat, Gerard Muller, Olivier Cha, Nadia Valin, Jean Nau, Pauline Campa, Benjamin Silbermann, Marc Bary, Pierre-Marie Girard, Karine Lacombe

**Affiliations:** 1 Inserm UMR-S707, Paris, France; 2 Université Pierre et Marie Curie, Paris, France; 3 Service de Maladies Infectieuses, Hôpital St Antoine, Assistance Publique- Hôpitaux de Paris (AP-HP), Paris, France; 4 Service d'Hépatologie, Hôpital St Antoine, AP-HP, Paris, France; 5 Unité de Santé Publique, Hôpital St Antoine, AP-HP, Paris, France; 6 Laboratoire de Virologie, Hôpital St Antoine, AP-HP, Paris, France; 7 Laboratoire St Marcel, Mairie de Paris, Paris, France; 8 Direction de l'Action Sociale, de l'Enfance et de la Santé, Mairie de Paris, Paris, France; 9 Centre de dépistage anonyme et gratuit (CDAG) du Figuier, Mairie de Paris, Paris, France; 10 Département des Examens Périodiques de Santé (DEPS), CPAM de Paris, France; 11 CDAG de Belleville, Paris, France; 12 Policlinique Baudelaire, Hôpital St Antoine, AP-HP, Paris, France; 13 Centre d'accueil, de soins et d'orientation, Médecins du Monde, Paris, France; 14 Unité de consultation et de soins ambulatoires (UCSA), Maison d'arrêt de la santé, Paris, France; 15 Centre Croix Rouge du Moulin Joly, Paris, France; Centers for Disease Control and Prevention, United States of America

## Abstract

**Background:**

In low hepatitis B virus (HBV)-prevalent countries, most HBV-infected persons are unaware of their status. We aimed to evaluate whether (i) previous HBV-testing, (ii) physicians decision to screen, and (iii) CDC's recommendations identified infected individuals and which risk-factor groups needing testing.

**Methods:**

During a mass, multi-center HBV-screening study from September 2010-August 2011, 3929 participants were screened for hepatitis B surface antigen (HBsAg), anti-HBs and anti-Hepatitis B core antibodies (anti-HBcAb). Questions on HBV risk-factors and testing practices were asked to participants, while participants' eligibility for HBV-testing was asked to study medical professionals.

**Results:**

85 (2.2%) participants were HBsAg-positive, while 659 (16.8%) had either resolved HBV infection or isolated anti-HBcAb. When comparing practices, HBV-testing was more likely to occur in HBV-infected participants if Centers for Disease Control and Prevention (CDC) recommendations were used (Sensitivity = 100%, 95%CI: 95.8–100) than physicians' discretion (Sensitivity = 87.1%, 95%CI: 78.0–93.4) or previous HBV-test (Sensitivity = 36.5%, 95%CI: 26.3–47.6) (p<0.0001). Nevertheless, many non-infected individuals would still have been screened using CDC-recommendations (Specificity = 31.1%, 95%CI: 29.6–32.6). Using multivariable logistic regression, HBsAg-positive status was significantly associated with the following: males, originating from high HBV-endemic region, contact with HBV-infected individual, without national healthcare, and intravenous-drug user (IDU). Of these risk-factors, physician's discretion for testing HBV was not significantly associated with participants' geographical origin or IDU.

**Conclusions:**

Missed opportunities of HBV-screening are largely due to underestimating country of origin as a risk-factor. Applying CDC-recommendations could improve HBV-screening, but with the disadvantage of many tests. Further development of HBV-testing strategies is necessary, especially before severe disease occurs.

## Introduction

Chronic hepatitis B virus (HBV) infection affects more than 350 million people worldwide, of whom 600,000 die each year of liver-related diseases associated with long-term infection [1], [2]. In Western countries, despite a relatively low prevalence and widely available access to vaccination, HBV remains one of the most frequent chronic infectious diseases, with a prevalence reaching 14 million in the European region compared to 9 million with hepatitis C virus (HCV) and 1.5 million with human immunodeficiency virus (HIV) [3].

Despite this high disease burden, few HBV infected patients are aware of their chronic infection. For instance, data have shown that 77% of HBV-infected persons in the European region [4], 65% in the United States [5] and up to 55% in France [6] are unaware of their status. These persons constitute a reservoir able to transmit HBV to other at-risk individuals and seek care at later stages of disease [7]. The high level of infection status unawareness can be explained by both the lack of knowledge in the general population or those at-risk [4], [8], [9], [10] and the lightness given by health care providers and policymakers with respect to its public health impact [3], [5].

As shown for HIV, identifying HBV-infected individuals through increased testing could be a necessary step towards decreasing the incidence of HBV [11]. However, testing recommendations are contentious across Europe. In France, there are no national guidelines, despite recent publication of the “National Plan for Hepatitis B and C” [12], and testing practices continue to be highly variable. The United States Centers of Disease Control and Prevention (CDC) do have official recommendations [7], in which individuals presenting with at least one of a large set of HBV risk-factors should be tested. To the best of our knowledge, the justification of such a strategy has never been evaluated in a diverse population, outside of specific risk-factor groups, such as clinics for sexually transmitted diseases (STD) [13], racial/ethnic minorities [14], immigrants [15], [16] etc. In order to assess these issues and suggest opportunities for improvement, we conducted a large, HBV-screening study among persons having different HBV transmission risk-factors in a wide range of healthcare settings where screening, prevention, and/or vaccination services are provided.

The general aim of the study herein was to determine the ability of various testing practices to identify infected and non-infected individuals. To this end, we first investigated if previous testing strategies reached HBsAg-positive persons and if these persons would have been tested by a physician. We then intended to evaluate whether identification of HBsAg-positive individuals was any different when applying CDC testing recommendations. Finally, we set out to compare what types of risk-factors distinguish persons with prior HBV-testing or testing via physician's discretion to those infected with HBV.

## Methods

### The OPTISCREEN-B program and study design

The OPTISCREEN-B program (www.optiscreenb.fr) was a multicenter, cross-sectional study in the Paris metropolitan region. Initially, we conducted an evaluation of several rapid tests that could be used to identify the presence of serological markers typically used in screening for chronic HBV infection. In the sub-study herein, we aimed to perform a comprehensive evaluation of screening practices among participants included in the program.

Ten centers, including a total of 75 study physicians, participated in the study. Healthcare centers were selected to represent those whose objectives included screening, prevention, and/or vaccination of diverse populations. More specifically, these centers provided one of the following services: free and anonymous sexually transmitted disease (STD) testing [Consultation de dépistage anonyme et gratuite (CDAG) Belleville, CDAG Figuier, CDAG St Antoine]; testing for the general population [Centre d'examens de santé de la Caisse Primaire d'Assurance Maladie (CPAM), Consultation Voyage St Antoine], immigrants and persons with low socioeconomic status (Centre de Santé rue au Maire, Médecins du Monde, Policlinique St Antoine, Croix-Rouge Moulin Joly), or incarcerated individuals [Unité de consultations et de soins ambulatoires (UCSA)]. All centers were required to be in the Paris metropolitan region so that HBV-infected patients could be referred to a single hospital (Saint-Antoine Hospital, Paris) for subsequent care.

The target screening population was persons potentially eligible for HBV-testing in healthcare centers, regardless of any HBV-testing recommendations. From September 2010 to August 2011, volunteers were asked to participate during their regular consultation if ≥18 years old and could be available for further contact. Since one major factor steering the decision to test in practice is complete certainty of prior HBV-infection or vaccination status, participants with a confirmed HBsAg-positive, anti-HBsAg antibody-positive, or anti-HBcAg antibody-positive test (requiring proof of result) were not included. This means that participants were still eligible if they declared having a previous test or vaccination but were unable to provide proof. Participants whose result was negative for all three HBV serological markers >6 months prior were also eligible. Importantly, no participant was excluded based on nationality, legal situation or access to government-provided healthcare. All participants provided written informed consent in their native language and the protocol was approved by the Hôtel-Dieu Hospital Ethics Committee (Paris, France) in accordance with the Helsinki Declaration.

### HBV risk-factor questionnaire

A questionnaire was administered by a trained clinical research assistant during a face-to-face interview with the participant. Questions were asked in lay terms on a variety of socio-demographic characteristics (age, sex, country of origin, parent's country of origin, education, employment status) and healthcare coverage. A section on risk-factors of HBV transmission was modeled from CDC testing recommendations [7] and a previous questionnaire developed by the Institut de Veille Sanitaire [6], as well as other potential risk-factors. HBV-endemicity of birth country was established according to WHO classification (prevalence of HBsAg-positive individuals): high (>8%), intermediate (2–8%), and low (<2%) [17]. Participants were also asked whether they had been vaccinated against HBV (verification of vaccination card was not performed).

### HBV serological battery and definition of HBV-infection status

**Figure 1:**
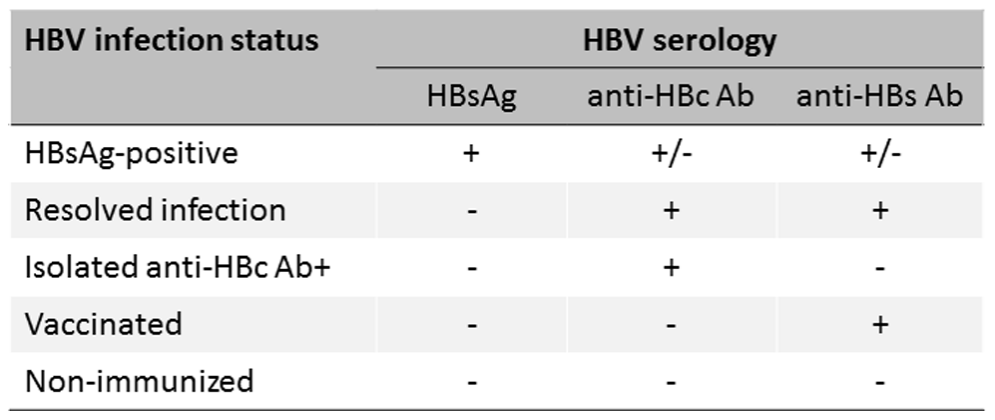
Definition of HBV infection status.

Full screening procedures have been detailed elsewhere [18] and are described in the supplementary file (Methods S1). HBV-infection status was based on serology and defined as in Figure 1. All HBsAg- and isolated anti-HBcAb positive individuals were contacted for a comprehensive medical exam at Saint-Antoine Hospital (Paris, France) in accordance with European recommendations [19].

### Assessing HBV-screening in practice

**Figure 2:**
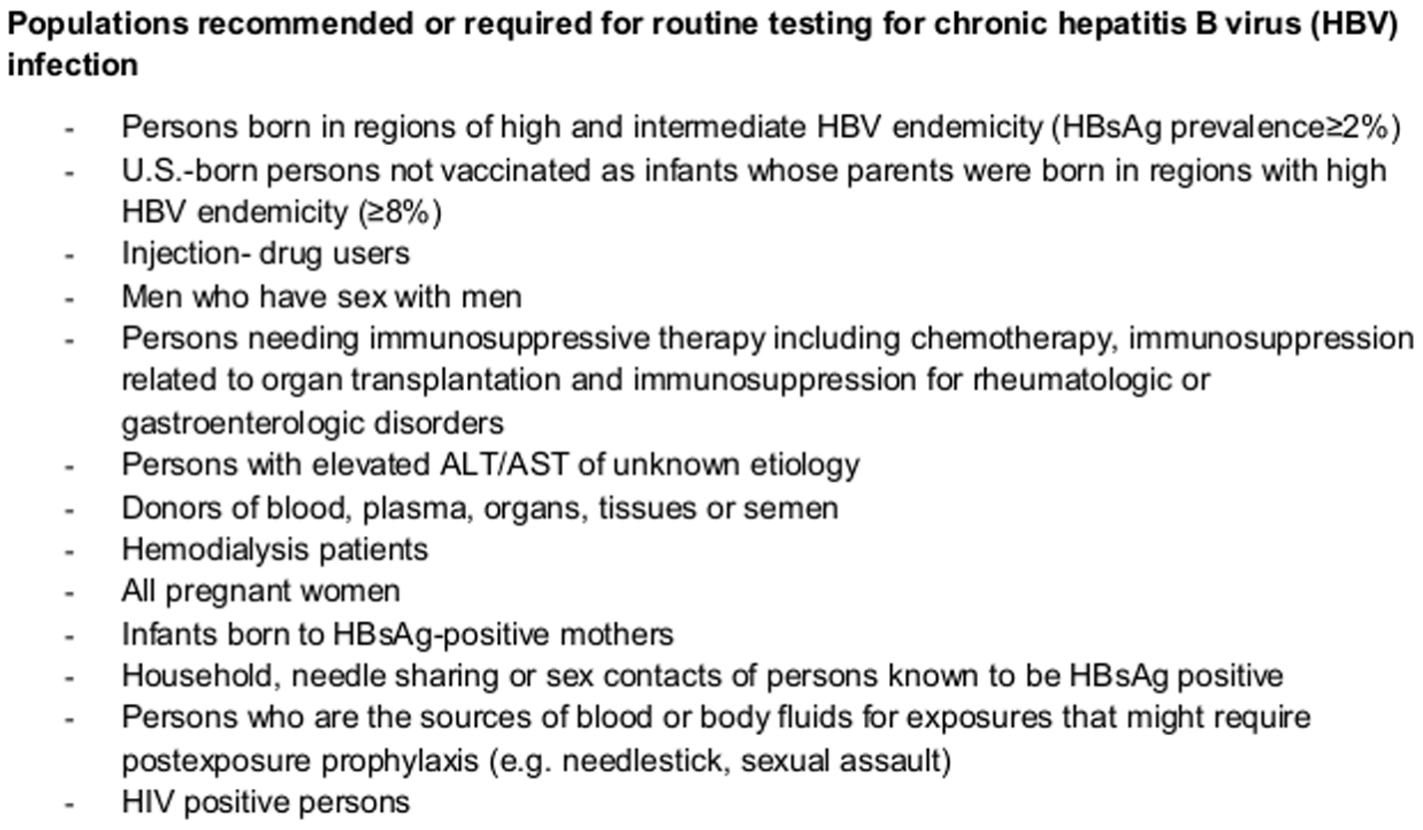
CDC recommendations for HBV screening.

Three major facets of HBV-testing were evaluated. First, patients were asked whether they had a previous HBV-test. Second, participating physicians were asked via separate questionnaire if they would have tested the participant regardless of inclusion in the OPTISCREEN-B study. Physicians were encouraged not to change their habitual HBV screening practices. Of note, no center-specific, regional, or national recommendations for HBV-screening were available. In addition, they were not given study questionnaires filled out by participants nor a checklist of CDC testing recommendations. Finally, we applied the CDC's HBV screening recommendations (Figure 2), determining that a given participant would be tested if they stated yes for any one of the listed risk-factors in their questionnaire [7].

### Statistical analysis

We determined the predictive capacity of (1) a previous HBV-test, (2) physicians' discretion, and (3) CDC recommendations in testing HBsAg-positive individuals. Sensitivity (Se), specificity (Sp), positive and negative predictive value (PPV and NPV, respectively) were estimated for each testing condition. Area under the receiving operator characteristic (AUROC) was used to estimate (Se+Sp)/2 and were compared between testing strategies using a test of equality of ROC areas from an algorithm developed by DeLong et al [20].

In order to evaluate risk-factors for a (1) a previous HBV-test, (2) physicians' discretion, and (3) HBsAg-positive status, we used random-effects logistic regression to determine the univariate association between risk-factors and each outcome (previous HBV-test, physicians' discretion for HBV test, and HBsAg-positive status) while accounting for within-center correlation. With a solely predictive objective in mind, a multivariable logistic model was then constructed in a forward-stepwise fashion, selecting risk-factors with a p-value <0.05 and testing their significance when added to the model using a likelihood ratio test. After developing the full model, covariates with p-values>0.05 were then removed one-by-one to obtain the final model. Specification error was tested by a link test for single-equation models without accounting for within-center correlation. Since many of the covariables were expected to be highly associated, tolerance (for any one variable, defined as 1-R^2^ determined when all other variables are regressed on that variable) and variance inflation factor (VIF, 1/tolerance) were used to determine the extent of collinearity. If all variables in the model are uncorrelated, both statistics are expected to be 1.

All statistical analyses were performed using STATA (v12.1, College Station, TX, USA) statistical software and significance was determined using a *p*-value<0.05.

## Results

### Study population

**Figure 3:**
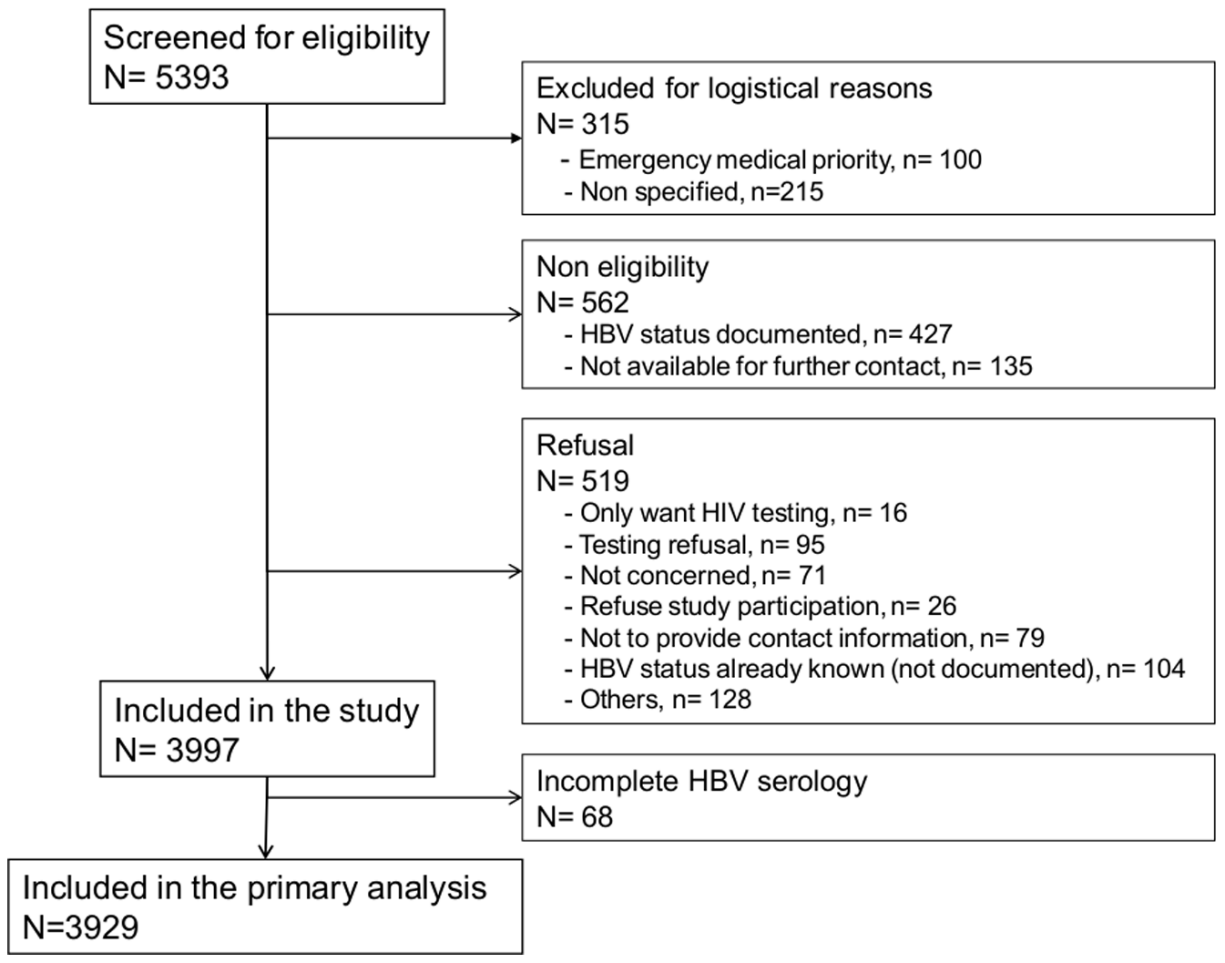
Study Flow Diagram.

Study flow diagram is provided in Figure 3. Among the 3929 individuals included in analysis, demographic characteristics and prevalence of various risk-factors for HBV transmission are presented in Table 1. Among the more commonly focused groups of individuals at risk for HBV-infection, 43.8% were born in a country of intermediate or high HBV-endemicity, 45.7% had >1 sexual partner 12-months prior, 23.1% had either no healthcare or healthcare assistance [currently using programs provided by the French government in the form of a Couverture Médicale Universelle (CMU) or an Aide Médicale d'Etat (AME)], 10.6% were men who have sex with men (MSM), and 0.6% were intravenous drug users (IDUs).

**Table 1:** Characteristics of the study population.

	*n* (%) (N = 3929)
Male	2196 (55.9)
Age* [N = 3928]	33 (25–45)
Claimed to have been vaccinated	1384 (35.2)
HBV prevalence of birth country	
Low (<2.0%)	2208 (56.2)
Intermediate (2.0–8.0%)	805 (20.5)
High (>8.0%)	916 (23.3)
Parents born in high HBV-endemic region [N = 3916]	1113 (28.4)
Traveled to high HBV-endemic region^1^	1155 (29.4)
Sought care in high HBV-endemic region	804 (20.5)
Health insurance plan	
Social security	3023 (76.9)
CMU^2^	227 (5.8)
AME^3^	177 (4.5)
Other	28 (0.7)
None	474 (12.1)
Surgical intervention	2437 (62.0)
Received transfusion before 1992	133 (3.4)
Received acupuncture	537 (13.7)
Received tattoos	530 (13.5)
Received piercing	1706 (43.4)
Close contact with an HBV + individual	304 (7.7)
Number of lifetime sexual partners	
0–1	690 (17.6)
2–9	1717 (43.7)
≥10	1522 (38.7)
>1 sexual partner within the last 12 months	1797 (45.7)
Men who have sex with men	416 (10.6)
Nasal drug-use	431 (11.0)
Intravenous drug-use	23 (0.6)
Long-term stay at a medical center	155 (4.0)
Previously incarcerated	201 (5.1)
Main clinical services provided at recruiting center	
General care and testing	1108 (28.2)
Free and anonymous STD testing	1859 (47.3)
Immigrant and low SES care	913 (23.2)
Care and testing for incarcerated individuals	49 (1.2)

*Median (IQR) given instead.

1 Period of stay was longer than 3 months.

2 Couverture médicale universelle, health insurance coverage that is given to persons living in precarious situations (i.e. unemployed, poverty, etc.).

3 Aide médicale d'état, health insurace generally given to immigrants without proper documentation.

Overall, there were 85 (2.2%) participants with positive HBsAg-serology, 528 (13.4%) with resolved HBV-infection, 131 (3.3%) with isolated anti-HBc Ab, 1710 (43.5%) vaccinated, and 1475 (37.5%) non-immunized. As shown in Table 2, many of the participants from high HBV-endemic countries had either positive HBsAg-serology or previous HBV-infection (58.4%). Of note, approximately one-third of participants were non-immunized across all HBV risk groups, with the exception of those from an intermediate HBV-endemic country (55.7% non-immunized).

**Table 2:** HBV-infection status within groups of risk-factors for HBV transmission.

HBV infection status	N	Zone of HBV-endemicity			>1 sexual partner within 12 mo.s	MSM	IDU
		Low	Int.	High			
	*n* = 3929	*n* = 2208	*n* = 805	*n* = 916	*n* = 1797	*n* = 416	*n* = 23
HBsAg-positive	85 (2.2)	1 (0.1)	15 (1.9)	69 (7.5)	18 (1.0)	1 (0.2)	2 (8.7)
Resolved infection	528 (13.4)	52 (2.4)	119 (14.8)	357 (39.0)	138 (7.7)	39 (9.4)	1 (4.4)
Isolated Anti-HBcAb	131 (3.3)	5 (0.2)	17 (2.1)	109 (11.9)	29 (1.6)	9 (2.2)	3 (13.0)
Vaccinated	1710 (43.5)	1368 (62.0)	206 (25.6)	136 (14.9)	1009 (56.2)	248 (59.6)	9 (39.1)
Non immunized	1475 (37.5)	782 (35.4)	448 (55.7)	245 (26.8)	603 (33.6)	119 (28.6)	8 (34.8)

HBV, hepatitis B virus; Int., intermediate; MSM, men who have sex with men; IDU, intravenous drug use.

### Performance of HBV-screening practices

#### Previous HBV-testing

Overall, 1199 (30.5%) participants claimed that they had been previously tested for HBV. Unsurprisingly, previous testing practices did not reach the majority of HBV-infected participants (Table 3), indicated by a poor Se (36.5%) and PPV (2.6%). This translates into 54/85 HBsAg-positive patients who would have benefited from HBV-testing and conversely 1168 persons who had a previous test without HBsAg. With an AUROC at 0.53 (95%CI: 0.48-0.58), previous testing did not appropriately identify HBsAg-positive participants.

**Table 3:** Performance of testing practices for HBsAg-positive individuals.

	N	HBsAg-positive *n (%)*		Classification probabilities (%)				
		Yes (*n* = 85)	No (*n* = 3844)	AUROC (95%CI)	Se	Sp	PPV	NPV
**Previous test**								
Yes	1199	31 (36.5)	1168 (30.4)	0.530 (0.478–0.582)	36.5	69.6	2.6	98.0
No	2730	54 (63.5)	2676 (69.6)					
**Test per physician**'**s discretion** 1								
Yes	2615	74 (87.1)	2541 (66.1)	0.605 (0.568–0.641)	87.1	33.9	2.8	99.2
No	1313	11 (12.9)	1302 (33.9)					
**Test per CDC recommendations** 2								
Yes	2735	85 (100)	2650 (68.9)	0.655 (0.648–0.663)	100	31.1	3.1	100
No	1194	0	1194 (31.1)					

1 If the study physician would have tested the participant for HBV per study center's protocol. One non-exposed participant had missing information and was excluded from this analysis.

2 If a participant would have been tested using the Center for Disease Control and Prevention (CDC) criteria for HBV screening [7].

#### HBV-testing as recommended by the physician

When asked if a participant needed HBV-testing, physicians recommended that 2615 (66.6%) should be tested. As shown in Table 3, physicians were likely to test HBV-infected individuals (Se = 87.1%). Nevertheless, 11/85 HBV infected patients would have missed the opportunity for HBV-testing and a large number of individuals would have been tested despite being non vaccinated or immunized (Sp = 33.9%).

#### Applying CDC guidelines for HBV-testing

When using CDC HBV screening recommendations [7] (Figure 2), all infected patients would have been tested for HBV (Se = 100%). Consequently, the area under the ROC was significantly higher for CDC recommendations (0.66) than physicians' discretion (0.61) or even previous HBV-test (0.53) (*p*<0.0001).

### Differentiating HBV-screening practices within HBV-infection groups

Since HBsAg-positive, resolved HBV-infection, and isolated anti-HBc Ab participants all had exposure to HBV, it would be extremely difficult to clinically differentiate the need for HBV testing between them. We conducted sensitivity analysis in which these three groups were compared to vaccinated or non-immunized patients. As shown in Table 4, there were no substantial differences in Se and Sp between the three categories, regardless of the HBV-screening practice considered. Among those not exposed to HBV; 975 (30.6%) had a previous test, 2074 (65.1%) were recommended screening by the physician, and 2015 (63.3%) would have been tested when applying CDC recommendations.

**Table 4:** Comparison of testing practices with respect to HBV-infection status.

	N	HBV-disease status *n (%)*			
		HBsAg positive	Resolved infection	Isolated Anti-HBc Ab	Non immunized/Vaccinated
**Previous test** 1		(*n* = 85)	(*n* = 528)	(*n* = 131)	(*n* = 3185)
Yes	1199	31 (36.5)	147 (27.8)	46 (35.1)	975 (30.6)
No	2730	54 (63.5)	381 (72.2)	85 (64.9)	2210 (69.4)
***Se/Sp*** *		***36.5/69.4***	***27.8/69.4***	***35.1/69.4***	***—***
***AUROC*** *		***0.529***	***0.486***	***0.523***	***—***
**Test per physician**'**s discretion** 1		(*n* = 85)	(*n* = 528)	(*n* = 131)	(*n* = 3184)
Yes	2615	74 (87.1)	376 (71.2)	91 (69.5)	2074 (65.1)
No	1313	11 (12.9)	152 (28.8)	40 (30.5)	1110 (34.9)
***Se/Sp*** *		***87.1/34.9***	***71.2/34.9***	***69.5/34.9***	***—***
***AUROC*** *		***0.610***	***0.530***	***0.522***	***—***
**Test per CDC recommendations** 2		(*n* = 85)	(*n* = 528)	(*n* = 131)	(*n* = 3185)
Yes	2735	85 (100.0)	505 (95.6)	130 (99.2)	2015 (63.3)
No	1194	0	23 (4.4)	1 (0.8)	1170 (36.7)
***Se/Sp*** *		***100/36.7***	***95.6/36.7***	***99.2/36.7***	***—***
***AUROC*** *		***0.684***	***0.662***	***0.680***	***—***

*Comparing HBV-status in column with non-immunized/vaccinated individuals.

1 If the study physician would have tested the participant for HBV per study center's protocol.

2 If a participant would have been tested using the Center for Disease Control and Prevention (CDC) criteria for HBV screening.[7].

### Contrasting determinants of HBV testing practices to HBsAg-positive status

Univariate analysis examining the determinants of individual HBV testing practices and HBsAg-positive serology are found in Table S1. In multivariable analysis (Table 5), the following groups were significantly associated with having been previously tested for HBV: claimed to have been vaccinated, parents from high HBV-endemic region, having a surgical intervention in a high HBV-endemic region, close contact with an HBV-infected individual, MSM, IDU, previously incarcerated, and having more than one sexual partner (lifetime or within the past 12 months). Of note, participants not currently under the national health plan were not significantly associated with a previous HBV test in the final model.

**Table 5:** Determinants of testing practices and HBsAg-positive status.

	Previous HBV-test N = 3916*		Eligible for HBV screening per physician's discretion^1^ N = 3914**		HBsAg-positive serology N = 3916*	
Risk-factor	OR (95% CI)	*p*	OR (95% CI)	*p*	OR (95% CI)	*p*
Female vs male	—	—	—	—	0.50 (0.31-0.82)	0.006
Age (per year)	—	—	0.98 (0.98-0.99)	<0.001	—	—
Claimed to have been vaccinated	2.50 (2.14–2.91)	<0.001	0.26 (0.21–0.30)	<0.001	0.36 (0.16–0.82)	0.02
Parents from high HBV-endemic region	1.62 (1.35–1.94)	<0.001	1.32 (1.08–1.61)	0.006	4.05 (2.17–7.55)	<0.001
Surgical intervention in high HBV-endemic region	1.31 (1.11–1.53)	0.001	—	—	—	—
Close contact with HBV + individual	1.82 (1.40–2.37)	<0.001	—	—	2.30 (1.23–4.30)	0.009
Men who have sex with men	3.03 (2.40–3.83)	<0.001	0.74 (0.58–0.96)	0.02	—	—
Intravenous drug-use	4.33 (1.44–13.03)	0.009	—	—	19.05 (3.18–114.06)	0.001
Previously incarcerated	1.52 (1.07–2.15)	0.02	1.59 (1.01–2.51)	0.04	—	—
Born in Int/High HBV-endemic region (>2.0%)	—	—	—	—	32.92 (4.37–248.08)	0.001
No health insurance plan^2^	—	—	1.78 (1.36–2.32)	<0.001	1.87 (1.04–3.36)	0.04
>1 lifetime sexual partner	2.04 (1.60–2.62)	<0.001	—	—	—	—

For each endpoint, all variables in the risk-factor column were used in the multivariable logistic regression model except for those with “—”. Further details on model construction are provided in Table S1.

*Thirteen patients were excluded from analysis as they had missing data on parent's geographical origin.

**Fifteen patients were excluded from analysis as 13 had missing data on parent's geographical origin, one had missing age, and one had missing physician's recommendation for testing.

1 Endpoint defined as if the study physician would have tested the participant for HBV per study center's protocol.

2 Includes participants with government assistance (CMU, AME) and “other” health insurance plans.

With respect to physician's discretion, participants with younger age, with parents from a high HBV-endemic region, previously incarcerated, with a health insurance plan other than the national social security, and with >1 sexual partner within the last 12 months were more likely to be tested for HBV in the multivariable model. Meanwhile, participants claiming to be vaccinated and MSM were significantly less likely to be tested by the physician.

Finally, independent determinants of HBsAg-positive serology were identified as follows: male gender, born in and having parents from a high HBV-endemic region, close contact with an HBV-infected individual, IDU, and having a health insurance plan other than national social security. As expected, those claiming to have been previously vaccinated were significantly less at risk of being HBsAg-positive (*p* = 0.02).

## Discussion

Our study is one of the largest and more recent examining HBV screening in a population with diverse HBV risk-factors performed in European country. We observed that individuals coming from intermediate and high HBV-endemic regions are in most need of HBV-testing and, while these individuals were more likely to be previously tested for HBV, they were not likely to be screened by the physician. More concerning was that individuals without national health insurance were at significant risk of having HBsAg-positive serology, yet were not likely to have had a previous HBV-test. The fact that physicians identified the need to test this specific population suggests that targeted campaigns are feasible. This study has particular interest in France, which hosts a large number of immigrants from intermediate to high HBV-endemic regions and is increasing funds for larger health coverage among them [21].

Certain caution should be given to the generalizability of our results. We observed a 2.2% prevalence of chronically HBV-infected individuals and 18.9% with past HBV-infection, corresponding to the prevalence criteria for moderate HBV-endemic countries. These estimates are almost two times higher than those observed in population-based studies in French metropolitan regions (HBsAg-positive: 0.8 and past HBV-infection: 10.8% [6]). These differences can be explained by a number of factors. First, we included a higher proportion of marginalized populations, such as immigrants, persons from lower socioeconomic status, and those without healthcare or needing healthcare assistance. Accordingly, there were roughly four-times more individuals born in high-HBV endemic countries in our study population (23.3%) than estimates from the Ile-de-France region (5.4%) [22]. It should be stated that these populations are usually underrepresented or ignored in prevalence estimates [6], [14], [23]. Second, our study population was mainly seeking care at free and anonymous STD testing centers and was also slightly younger than the general population (median age: 33 versus 39, respectively) [24]. Third, we excluded any patient with a confirmed, positive test for HBsAg, anti-HBsAg Ab and/or anti-HBcAb. The reasons for this exclusion criterion were driven by our clinical experience. Taken together, our intent was not to represent the general population in what might be considered a “universal” screening campaign, rather those individuals who could be potentially eligible for HBV testing specifically in healthcare settings with screening, prevention, and/or vaccination services.

Judging from previous HBV-testing, it would appear that screening practices had been based on a wide-range of at-risk populations, but a proportionally higher amount of testing was performed among IDU, MSM, and persons with more than one sexual partner. When comparing these results to HBsAg-positive individuals, IDU and those without health insurance were certainly at need for screening, but previous testing practices would seem, as previously suggested [13], to overemphasize sexual practice as a risk-factor.

We also clearly demonstrate that CDC recommendations allow for a more efficient testing strategy than via physicians' discretion or previous testing strategies. When looking at individual classification probabilities, it comes as no surprise that CDC criteria, with thirteen extremely diverse criteria, had perfect sensitivity in our study. In addition, a major advantage of the CDC recommendations is its capacity to practically eliminate missed opportunities of HBV-testing (i.e. patients needing testing who were not tested), contrary to physician's discretion where 12.9% of HBsAg-positive patients would not have been tested for HBV. However, screening recommendations such as these come with the disadvantage of many tests, the extent of which has not yet been formally evaluated. From our data, the specificity was remarkably poor, putting into question whether the current recommendations are too overreaching. Still, the specificity of physician's discretion for HBV-testing was very similar to CDC recommendations, implying that there could be some benefit to applying these recommendations.

Nevertheless, one important consideration when testing for HBV is the complexity of HBV infection itself. HBV-exposed individuals (i.e. HBsAg-positive, resolved infection, isolated HBcAb-positive) all share common transmission risk-factor patterns, yet not all are infected. We observed that the Se and Sp of these three infection groups did not change when compared to nonimmunized/vaccinated individuals, suggesting that all strategies share the common difficulty of distinguishing HBV-infected individuals. Our findings highlight that recommendations should address this particular issue.

Several other limitations need to be addressed in our study. First, CDC recommendations for HBV screening were discussed during investigator meetings and the on-site presence of OPTISCREEN-B study personnel was fairly obvious. Even though we explicitly asked physicians if they would have tested each individual for HBV despite participating in the study, we cannot rule out any potential observation bias that physicians were more inclined to test for HBV. Second, since this was a convenience sample from a previous validation study, we did not incorporate any specific sampling scheme in the study design and therefore our results were not corrected to represent the general population using weighted estimates. Furthermore, as an ancillary objective was to comprehensively evaluate the clinical status of all HBV-infected patients, we chose not to include an overly large catchment area so that all patients could be followed at a single center. Third, there were certain issues concerning the models. Many risk-factors were highly correlated, especially between parents from high endemic countries, no health insurance plan, and born in intermediate/high HBV-endemic regions. The collinearity of these variables had most likely a modest impact on the models, as determined by the variance inflation factors. Model overfitting could have also occurred when predicting risk-factors for HBsAg-positive status, in particular for IDU and born in intermediate/high HBV-endemic regions. However, due to their importance, we decided not to exclude them from the model. Finally, despite the majority of exclusions being due to prior confirmed tests and not for logistical reasons, there was a notable percentage of patients refusing participation in the study. However, it should be stated that participation rates in our study were similar, if not higher, than previous studies.[13], [25], [26] Notwithstanding these limitations, this study does have several important features, including diverse recruiting centers, large number of participating physicians, broad inclusion criteria, and complete HBV serology.

In conclusion, although our study population may not be fully representative of the general population, we demonstrate that more emphasis needs to be placed on testing individuals from intermediate to high HBV-endemic countries, IDUs, and limited access to health care; but not necessarily those with increased sexual activity. CDC recommendations may provide additional benefit when compared to physicians' discretion, yet both have the disadvantage of many tests. Future research should aim at increasing awareness of HBV-infection status with the use of rapid HBV testing, whose ease of use could meet the need for an immediate testing result. These testing strategies are reliable [18] and could increase linkage to care, thereby reducing lost to follow-up and possibly increasing cost-effectiveness. Although our study focused on more successful strategies to screen infected individuals and ignored the clinical implications of a negative test, it should be further evaluated whether testing can be used as a gateway for HBV vaccination among non-immunized individuals.

## Supporting Information

Table S1 Univariate analysis for testing practices and HBsAg positive status.
(DOCX)

Methods S1 Complete HBV serological battery.
(DOCX)
