# Supplementary material for: Current State of and Needs for Hepatitis B Screening: Results of a Large Screening Study in a Low-Prevalent, Metropolitan Region
Source: PLoS One. 2014 Mar 24;9(3):e92266. doi: 10.1371/journal.pone.0092266 (PMC3963888; doi:10.1371/journal.pone.0092266)
Supplement: Table S1 — Univariate analysis for testing practices and HBsAg positive status. (DOCX) [file pone.0092266.s001.docx]

**TABLE S1. Univariate analysis for testing practices and HBsAg positive status**

| **Risk-factor** | | **Previous HBV-test*** | | **Eligible for HBV screening per physician’s discretion**^1^** | | **HBsAg-positive serology***** | |  |
| --- | --- | --- | --- | --- | --- | --- | --- | --- |
|  |  | N=3929 | | N=3928 | | N=3929 |  | |
|  |  | OR (95% CI) | *p* | OR (95% CI) | *p* | OR (95% CI) | *p* | |
| Female vs male | | 0.89 (0.77-1.02) | 0.09 | 1.05 (0.91-1.22) | 0.5 | **0.52 (0.32-0.85)** | **0.009** | |
| Age (per year) | | 1.00 (0.99-1.01) | 0.3 | **0.99 (0.98-0.99)** | **<0.001** | 0.99 (0.97-1.00) | 0.13 | |
| Claimed to have been vaccinated | | **2.74 (2.37-3.18)** | **<0.001** | **0.25 (0.21-0.30)** | **<0.001** | **0.30 (0.13-0.66)** | **0.003** | |
| Parents from high endemic region | | **1.29 (1.09-1.54)** | **0.003** | **1.53 (1.27-1.84)** | **<0.001** | **6.16 (3.35-11.31)** | **<0.001** | |
| Traveled to high endemic region^2^ | | 1.17 (0.99-1.39) | 0.07 | **1.44 (0.20-1.72)** | **<0.001** | **4.56 (2.55-8.15)** | **<0.001** | |
| Received care in high endemic region | | **1.33 (1.10-1.62)** | **0.004** | **1.40 (1.14-1.73)** | **0.001** | **2.72 (1.63-4.54)** | **<0.001** | |
| Surgical intervention | | **1.37 (1.18-1.59)** | **<0.001** | **0.79 (0.67-0.92)** | **0.003** | **0.51 (0.32-0.82)** | **0.005** | |
| Transfusion before 1992 | | 1.28 (0.87-1.86) | 0.2 | 0.71 (0.47-1.07) | 0.10 | 0.55 (0.13-2.30) | 0.4 | |
| Acupuncture | | **1.25 (1.02-1.52)** | **0.03** | 0.84 (0.68-1.04) | 0.11 | 1.34 (0.70-2.57) | 0.4 | |
| Tattoos | | **1.27 (1.04-1.55)** | **0.02** | **1.25 (1.00-1.57)** | **0.049** | 0.88 (0.43-1.80) | 0.7 | |
| Piercing | | 1.11 (0.96-1.27) | 0.16 | 1.15 (0.99-1.33) | 0.08 | 0.71 (0.44-1.13) | 0.15 | |
| Close contact with HBV+ individual | | **2.01 (1.58-2.58)** | **<0.001** | 0.78 (0.59-1.04) | 0.09 | **1.89 (1.04-3.44)** | **0.04** | |
| Men who have sex with men | | **3.45 (2.76-4.31)** | **<0.001** | **0.55 (0.43-0.70)** | **<0.001** | 0.26 (0.03-1.91) | 0.18 | |
| Nasal drug-use | | **1.60 (1.29-1.97)** | **<0.001** | 0.89 (0.70-1.14) | 0.4 | 0.21 (0.03-1.58) | 0.13 | |
| Intravenous drug-use | | **8.34 (3.00-22.64)** | **<0.001** | 0.41 (0.16-1.09) | 0.08 | **6.42 (1.30-31.80)** | **0.02** | |
| Long-term stay at a medical center | | 1.00 (0.70-1.44) | 0.9 | 0.82 (0.57-1.18) | 0.3 | 0.82 (0.25-2.67) | 0.7 | |
| Previously incarcerated | | **1.52 (1.08-2.14)** | **0.02** | **1.53 (1.00-2.35)** | **0.05** | 0.56 (0.20-1.62) | 0.3 | |
| HBV-prevalence of birth region | |  |  |  |  |  |  | |
|  | Low (<2.0%) | **1.00** |  | **1.00** |  | **1.00** |  | |
|  | Intermediate (2.0-8.0%) | **1.34 (1.08-1.66)** | **0.007** | **1.90 (1.52-2.39)** | **<0.001** | **126.93 (16.96-949.96)** | **<0.001** | |
|  | High (>8.0%) | **0.96 (0.78-1.17)** | **0.7** | **1.36 (1.11-1.67)** | **0.003** | **36.47 (4.72-281.73)** | **0.001** | |
| Health insurance plan | |  |  |  |  |  |  | |
|  | Social security | **1.00** |  | **1.00** |  | **1.00** |  | |
|  | CMU^3^ | **0.82 (0.58-1.14)** | **0.2** | **1.86 (1.31-2.65)** | **0.001** | **2.25 (0.90-5.65)** | **0.09** | |
|  | AME^4^ | **1.18 (0.80-1.74)** | **0.4** | **1.94 (1.27-2.96)** | **0.002** | **2.71 (1.05-7.02)** | **0.04** | |
|  | Other | **0.56 (0.22-1.40)** | **0.2** | **1.82 (0.67-4.91)** | **0.2** | ****** |  | |
|  | None | **0.71 (0.52-0.99)** | **0.04** | **3.22 (2.18-4.75)** | **<0.001** | **6.60 (3.10-14.02)** | **<0.001** | |
| Nb of life-time sexual partners | |  |  |  |  |  |  |  |
|  | 0-1 | **1.00** |  | **1.00** |  | 1.00 |  | |
|  | 2-9 | **1.92 (1.49-2.47)** | **<0.001** | **1.01 (0.81-1.26)** | **0.9** | 1.15 (0.68-1.95) | 0.6 | |
|  | ≥10 | **3.23 (2.51-4.17)** | **<0.001** | **0.75 (0.59-0.94)** | **0.01** | 0.63 (0.31-1.28) | 0.2 | |
| >1 sexual partner within 12 mo.s | | **1.46 (1.23-1.72)** | **<0.001** | 0.99 (0.82-1.19) | 0.9 | 0.69 (0.38-1.27) | 0.2 | |

^1^Endpoint defined as if the study physician would have tested the participant for HBV per study center’s protocol.

^2^Period of stay was longer than 3 months.

^3^Couverture médicale universelle, health insurance coverage that is given to persons living in precarious situations (i.e. unemployed, poverty, etc.).

^4^Aide médicale d’état, health insurance generally given to immigrants without proper documentation.

OR in bold were included in a forward-stepwise selection of variables:

*During forward-stepwise selection, the following variables were no longer below the pre-specified *p*-value threshold and hence excluded from the model: received care in a high endemic region (*p*=0.060), acupuncture (*p*=0.061), HBV-prevalence of birth region (*p*=0.123), and >1 sexual partner within 12 mo.s (*p*=0.251). After construction of the full model, we removed the following variables because they were no longer below the pre-specified threshold: tattoos (*p*=0.381), and nasal drug-use (*p*=0.088). Using a link test for single-equation models, the linear predicted value squared was not significant (*p*=0.524), suggesting that the model is properly specified. In the final model, the respective tolerance and variance inflation factor for each variable were as follows: claimed to have been vaccinated (0.93, 1.07), parents from high endemic region (0.90, 1.11), surgical intervention (0.95, 1.06), close contact with HBV+ individual (0.99, 1.01), men who have sex with men (0.95, 1.06), intravenous drug-use (0.98, 1.02), previously incarcerated (0.98, 1.02), >1 lifetime sexual partner (0.93, 1.08).

**During forward-stepwise selection, the following variables were no longer below the pre-specified *p*-value threshold and hence excluded from the model: traveled to high endemic region (*p*=0.094), received care in high HBV-endemic region (*p*=0.565), received surgical intervention in high HBV-endemic region (*p*=0.698), tattoos (*p*=0.117), and >1 lifetime sexual partner (*p*=0.121). After construction of the full model, we removed the following variables because they were no longer below the pre-specified threshold: moderate/high HBV-prevalence of birth region (*p*=0.073). Using a link test for single-equation models, the linear predicted value squared was not significant (*p*=0.738), suggesting that the model is properly specified. In the final model, the respective tolerance and variance inflation factor for each variable were as follows: age (0.98, 1.02), claimed to have been vaccinated (0.92, 1.09), parents from high HBV-endemic region (0.83, 1.20), previously incarcerated (0.98, 1.02), no health insurance plan (0.80, 1.25).

***During forward-stepwise selection, the following variables were no longer below the pre-specified *p*-value threshold and hence excluded from the model: traveled to a high endemic region (*p*=0.401), received care in high HBV-endemic region (*p*=0.783), surgical intervention (*p*=0.098). Using a link test for single-equation models, the linear predicted value squared was not significant (*p*=0.560), suggesting that the model is properly specified. In the final model, the respective tolerance and variance inflation factor for each variable were as follows: gender (0.99, 1.01), claimed to have been vaccinated (0.92, 1.08), parents from high HBV-endemic region (0.73, 1.37), close contact with HBV+ individual (0.99, 1.01), intravenous drug-use (0.99, 1.01), born in int/high HBV-endemic regions (0.63, 1.58) and no health insurance plan (0.70, 1.42).
