# Supplementary material for: Current State of and Needs for Hepatitis B Screening: Results of a Large Screening Study in a Low-Prevalent, Metropolitan Region
Source: PLoS One. 2014 Mar 24;9(3):e92266. doi: 10.1371/journal.pone.0092266 (PMC3963888; doi:10.1371/journal.pone.0092266)
Supplement: Methods S1 — Complete HBV serological battery. (DOCX) [file pone.0092266.s002.docx]

**METHODS S1**

***Complete HBV serological battery***

Briefly, a whole blood sample of approximately 10 ml was drawn at the time of screening visit. Qualitative results for hepatitis B surface antigen (HBsAg), anti-HBs antibodies (anti-HBs Ab), and anti-hepatitis B core antibodies (anti-HBc Ab) were determined from extracted serum via a commercially-available enzyme-linked immuno-assay (ELISA) (MONOLISA AgHBS Ultra; anti-HBs plus, anti-hepatitis B core antibody-anti-HBc- plus, BIORAD, Hercules, USA). All HBsAg-positive samples were confirmed by a seroneutralization assay (BIORAD, Hercules, USA) and/or by HBsAg quantification (ARCHITECT HBsAg Chemoluminescence; Abbott Laboratories, Rungis, France). All inconclusive anti-HBs Ab results (at detection threshold) were considered negative.
